# Rapid DNA origami nanostructure detection and classification using the YOLOv5 deep convolutional neural network

**DOI:** 10.1038/s41598-022-07759-3

**Published:** 2022-03-09

**Authors:** Matthew Chiriboga, Christopher M. Green, David A. Hastman, Divita Mathur, Qi Wei, Sebastían A. Díaz, Igor L. Medintz, Remi Veneziano

**Affiliations:** 1 Center for Bio/Molecular Science and Engineering Code 6900, U.S. Naval Research Laboratory, Washington, DC 20375 USA; 2 Department of Bioengineering, Volgenau School of Engineering, George Mason University, Fairfax, VA 22030 USA; 3 National Research Council, Washington, DC 20001 USA; 4 Fischell Department of Bioengineering, A. James Clark School of Engineering, University of Maryland, College Park, MD 20742 USA; 5 College of Science, George Mason University, Fairfax, VA 22030 USA

**Keywords:** DNA, Nanobiotechnology, Nanostructures

## Abstract

The intra-image identification of DNA structures is essential to rapid prototyping and quality control of self-assembled DNA origami scaffold systems. We postulate that the YOLO modern object detection platform commonly used for facial recognition can be applied to rapidly scour atomic force microscope (AFM) images for identifying correctly formed DNA nanostructures with high fidelity. To make this approach widely available, we use open-source software and provide a straightforward procedure for designing a tailored, intelligent identification platform which can easily be repurposed to fit arbitrary structural geometries beyond AFM images of DNA structures. Here, we describe methods to acquire and generate the necessary components to create this robust system. Beginning with DNA structure design, we detail AFM imaging, data point annotation, data augmentation, model training, and inference. To demonstrate the adaptability of this system, we assembled two distinct DNA origami architectures (triangles and breadboards) for detection in raw AFM images. Using the images acquired of each structure, we trained two separate single class object identification models unique to each architecture. By applying these models in sequence, we correctly identified 3470 structures from a total population of 3617 using images that sometimes included a third DNA origami structure as well as other impurities. Analysis was completed in under 20 s with results yielding an F1 score of 0.96 using our approach.

## Introduction

Rapid progress in the field of artificial intelligence (AI) and the subfield machine learning (ML) has led to a diverse landscape of deep learning (DL) algorithms. Various ML subfields apply DL algorithms to specific problems. For example, computer vision (CV) is a ML subfield which encompasses the application of ML algorithms to develop intelligent software with human-like visual recognition and comprehension capabilities. Commonly, this CV visual recognition is done using convolutional neural networks (CNNs). A prevailing CV tool, CNNs first garnered popularity for their ability to recognize pixel patterns in images to identify numerical digits^1,2^. Modern deep CNNs (dCNNs) have been shown to be highly effective in even more complex image processing and image analysis tasks. These dCNNs aim to tackle problems of image classification, object detection, semantic segmentation, image generation, and scene understanding^3–8^. Until recently, limited access to expensive hardware, in-house datasets, proprietary software libraries, and technical implementation expertise has limited dCNNs to use by commercial and industrial manufacturers. For example, commercial uses include facial recognition to unlock phones or autonomous vehicle development using cameras to recognize street signs. Industrial manufacturers deploy dCNN algorithms to identify product surface defects in quality control image pipelines^9–12^. Each use involves analyzing static images or audio/video data to identify objects of interest rapidly and *en masse*. Fortunately, in recent years, high-performance cloud computing platforms and pushes toward open-source software have increased the access of modern dCNNs to a wider community^13^. dCNNs are now poised to break into new areas of research, engineering, and reconnaissance, especially in tedious tasks constrained by limits in human attentiveness and fatigue. For instance, the lowered barrier to entry now empowers dCNNs to be applied where rapid microscopic object detection is not an indispensable or critical component, but instead a potentially rate-limiting step with potential for optimization^14,15^. In these cases, dCNNs could substantially improve sample throughput, analysis consistency, energy/resource allocation, and experimental replication by others.

One such area which would greatly benefit from increased access to modern CV techniques is the field of DNA nanotechnology. This field leverages the intrinsic biochemical and physical properties of DNA to form complex 2- and 3-dimensional nanoscale architectures. Using modern structural design and assembly techniques in conjunction with open-source software, the fabrication of nearly any arbitrary nanoscale geometry becomes possible^16–20^. Since these structures are synthesized on the molecular scale, the total number of structures in a given sample is proportional to Avogadro’s number. Exploiting the sequence predictability of the Watson–Crick base pair rules, DNA scaffolds have been deployed as a functional material in a diverse range of applications. For example, the unique ability of DNA to controllably hybridize and dissociate has enabled the creation of dynamic nanostructures and DNA robots responsive to molecular stimuli^21,22^. Furthermore, the large library of conjugation chemistries and the one-pot self-assembly of DNA nanostructures have allowed DNA to be coupled with a variety of functional groups to create multifunctional materials. Common DNA modifications can include metallic nanoparticles, enzymes, small molecules, and fluorescent dyes^23–27^. Additionally, the nontoxic nature of DNA lends itself well for application in the biomedical and drug delivery sectors^28–31^.

Atomic force microscopy (AFM) and transmission electron microscopy (TEM) have become the most common modalities to assay the assembly and structural fidelity of a given DNA architecture or sample^32,33^. The advantage of these two measurement modalities lies in their ability to resolve distances below the Rayleigh-Abbe diffraction limit of visible light^34^. DNA-PAINT, a fluorescence-based super-resolution microscopy approach, is daily expanding in its use, though imaging and analysis is often restricted to a handful of modified sites rather than an entire structure^35,36^. Such super-resolution techniques are nonetheless valuable because they provide a means to quantify the availability of addressable sites and enable single-molecule studies of interactions between biomolecules on DNA nanostructures^37,38^. Conveniently, all these microscopies enable the acquisition of large image sets for ensemble statistical analysis, with each image potentially containing thousands of structures; for example, a 20 × 20 μm^2^ AFM image of DNA origami on a mica substrate can easily contain more than 10,000 origami assemblies. Determining assembly fidelity in such samples requires that multiple images and samples be examined to get a true reflection of the entire ensemble. Compared to other microscopy communities, the bio-AFM community has relatively few open source and freely available software packages enabling the automated identification and classification of DNA nanostructures^39^. As a result, AFM often requires manual annotation for analysis, and the sheer number of structures present in large image sets precludes manual annotation and limits analyses to partial data sets. Moreover, manual annotation is typically inconsistent between annotators and even between multiple annotations performed by a single annotator due to fatigue. For example, as of April 2021, the Code of Federal Regulations Flow Cytometry standard mandates that no individual can examine greater than 100 pathological specimen slides under a microscope in a 24-h period due to fatigue^40,]^^41,42^. This cost in accuracy then impacts the downstream reproducibility and significance of experimental results.

Here, we address the issue of manual structure annotation by outlining a protocol for the rapid automation of DNA structure detection and classification in raw AFM image data. The method is a generalized approach and thus should translate to other high-resolution microscopies and/or other DNA structural geometries. We provide a multi-step outline for data acquisition, data preparation, neural network model configuration, model training, making predictions (inference), prediction analysis, and data curation. We utilize the You Only Look Once (YOLO) object detection CNN framework^43–46^ and specifically, the YOLOv5 PyTorch implementation of this framework. To demonstrate the power of this protocol, we examine three different DNA architectures each with distinct geometries. Source images of the three distinct DNA origami nanostructures were obtained from an in-house experimental image repository. We applied data augmentation/transformation functions to the source images to create more diverse and regularized training sets. Using the augmented training data, we subsequently fit (train) YOLOv5s models to DNA nanostructures based on triangle and breadboard geometries, with a nanotube nanostructure acting as a non-identified control. Using these trained models, we were able to accurately identify DNA nanostructures in a separate set of AFM images. Statistical analysis of binary classification was used to quantify each model’s performance and demonstrate high geometric precision and robust structure recall. We observed each model to perform with an F1 score > 0.93. Then, by employing each classifier in sequence, we were able to achieve triangle-breadboard multiclass identification. Although we only exploit two classes represented by the two identified DNA structures, this protocol is, in theory, able to be extrapolated to identify an arbitrarily high number of classes. The large input parameter space of dCNNs and the feature extraction of the YOLO framework enables the network to generalize the patterns of each structure. Consequently, this detection method would potentially be robust and therefore advantageous in instances of datasets with non-uniform experimental conditions.

## Results

### Data preparation

**Figure 1:**
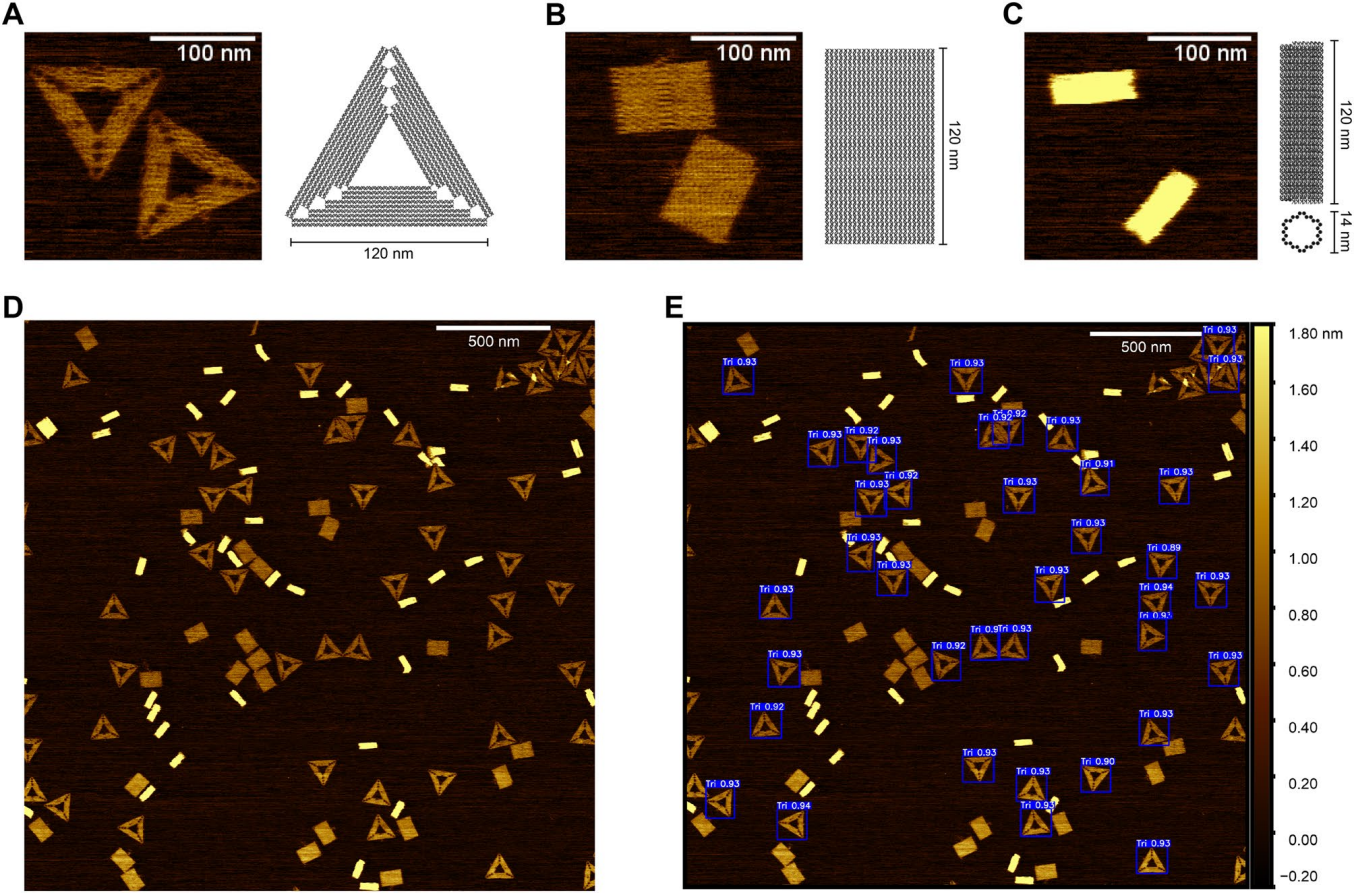
Structure schematics and sample image pre- and post-detection. Image examples of the three structures used, along with accompanying ribbon diagrams. Examples include: (**A**) the sharp triangle, (**B**) breadboard, and (**C**) nanotube architectures. (**D**) Selected example of a test image pre-detection containing all three architectures. (**E**) Selected example of the test image post-triangle detection. The blue bounding box masks are output by the YOLOv5s model representing identified triangles. Size bars in panel (**A**–**C**) are 100 nm. Size bars in (**D**,**E**) are 500 nm.

**Figure 2:**
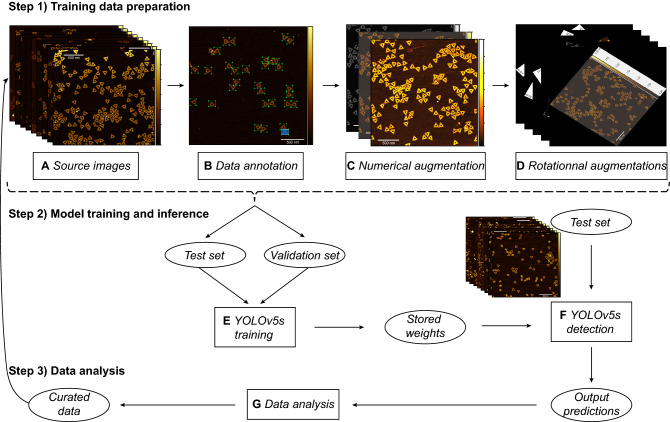
Procedural flow chart of data processing and analysis. Steps taken to configure and utilize a YOLOv5s model. **Step 1**, data preparation beginning with (**A**) image capture to form the source dataset. (**B**) The source data structures are annotated. (**C**) The resulting images are input to numerical augmentation functions. The numerically augmented images are input to (**D**) a rotational augmentation function five times. **Step 2** outlines working with the YOLOv5s model. The training and validation data sets are input to (**E**) fit a YOLOv5s CNN which is exported to a PyTorch weights file. These weights are used to make predictions on the test set. The YOLOv5 detection function (**F**) outputs predictions based on the input weights. Lastly, **step 3** outlines what happens to the data output from the YOLOv5s model. In (**G**) the statistics of the YOLOv5s predictions are calculated to measure performance. Once erroneous predictions are corrected, the curated data can be combined with the source data for future training(s).

For a more detailed description of the methods used please see “Materials and Methods” and the *Supporting Information* (SI) sections (vide infra). Two distinct DNA origami geometries were selected for YOLO identification, namely the sharp triangle^17^ and rectangular “breadboard”^47^, as well as a negative control origami structure, a 30-helix nanotube^48^ (Fig. 1). These geometries are prototypical, used in a variety of applications, and have been relatively well characterized^49^. The source AFM images represented moderately diverse experimental and AFM conditions based on the general procedures described in the Methods section. Once captured, the source images represent the first step in the training data preparation process (Fig. 2). For the triangle architecture, our source data consisted of 10 images with 818 annotated triangle data points. The breadboard source dataset consisted of 5 images with 176 annotated breadboard data points. The source data was input to the augmentation functions as described in the Methods and outlined in Fig. 2. Following data preparation, the triangle training set consisted of 200 images with 16,360 annotated data points while the validation set consisted of 40 images with 3,272 data points. For the breadboard architecture, the training set consisted of 100 images with 3,520 annotated data points and the validation set consisted of 20 images with 704 data points. Following data preparation, the next steps focus on model training and inference (see Fig. 2).

Prior to training and inference, we captured an additional set of images referred to as the test set (see Fig. 2), which remained isolated from the model during training. The purpose of the test set is to function as a separate dataset on which the model can make predictions, by inference. Our test set is composed of 36 images containing 1,986 triangles and 1,621 breadboards. These data points, obtained by manual curation of model predictions, are known as the ground truth data, which we use for comparison with the predictions generated by our YOLOv5 models. The test set is decomposed into subsets that target specific populations of interest. These subsets target different architecture distributions (homogenous and heterogeneous), different magnifications (low, medium, and high), and different intra-image clustering (full and 1 quadrant) (Supplementary Fig. 7). The exact image test set magnification values for each image and classifier can be seen in (Supplementary Table 2). The ground truth annotations of these subsets, respectively, are outlined in Table 1.

**Table 1:** Outline of each test subset including the subset type, number of images, geometry in question, number of ground truth annotations, and number of predictions.

Test subset	# of Images	Geometry	# Annotations	# Predictions
Homogenous triangle	14	Triangle	1,355	1,357
		Breadboard	15	10
		Combined	1,370	1,367
Homogenous Breadboard	14	Triangle	–	–
		Breadboard	829	847
		Combined	829	847
Heterogeneous	8	Triangle	631	639
		Breadboard	777	739
		Combined	1,408	1,378
Low Magnification	8	Triangle	985	995
		Breadboard	1,156	1,115
		Combined	2,141	2,110
Medium Magnification	18	Triangle	1,001	1,001
		Breadboard	253	222
		Combined	1,254	1,223
High Magnification	10	Triangle	–	–
		Breadboard	212	259
		Combined	212	259
Clustered Full	10	Triangle	656	654
		Breadboard	176	215
		Combined	832	869
Clustered 1Quadrant	10	Triangle	94	94
		Breadboard	36	44
		Combined	130	138
Full Dataset	36	Triangle	1,986	1,996
		Breadboard	1,621	1,596
		Combined	3,607	3,592

### Neural network training

The training and validation data were directly input to the YOLOv5 training function in order to fit a YOLOv5s object detection model as described in the Methods (Fig. 2). In terms of model configuration, the YOLOv5 codebase has several prewritten model structures available to choose from (Supplementary Table 1). Generally speaking, the prewritten YOLOv5 models trade speed for accuracy. In the interest of broad accessibility and applicability, we chose the YOLOv5s model as it can be easily executed on freely available cloud computing platforms such as Google Colaboratory. In total, the amount of data used to fit the triangle model was approximately 820 MB. Using this data, training took approximately 3 h and 8 min on a V100-SXM2-16 GB GPU. Comparatively, the breadboard data summed to about 720 MB and took approximately 2 h and 4 min to train on a P100-PCIe-16 GB GPU. The YOLOv5 training function automatically exports the learned weights and biases into a corresponding PyTorch weights file along with available metadata (Fig. 2). See Supplementary Fig. 8 for training loss convergence plots.

### Inference and performance analysis

**Figure 3:**
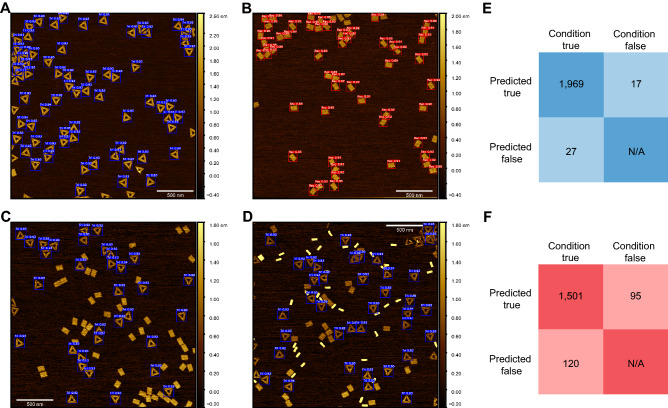
Model activation in response to geometric diversity. Selected images of different sample populations post-detection. (**A**) Homogenous triangle population post-triangle detection. (**B**) Homogenous breadboard population post-breadboard detection. (**C**) Heterogeneous sample population of triangle + breadboard post-triangle detection. (**D**) Heterogeneous sample population of triangle + breadboard + nanotube post-triangle. (**E**) Confusion matrix representing predictions made by the triangle classifier on the entire test set. (**F**) Confusion matrix representing predictions made by the breadboard classifier on the entire test set. Scale bars in panel (**A**–**D**) are 500 nm.

**Figure 4:**
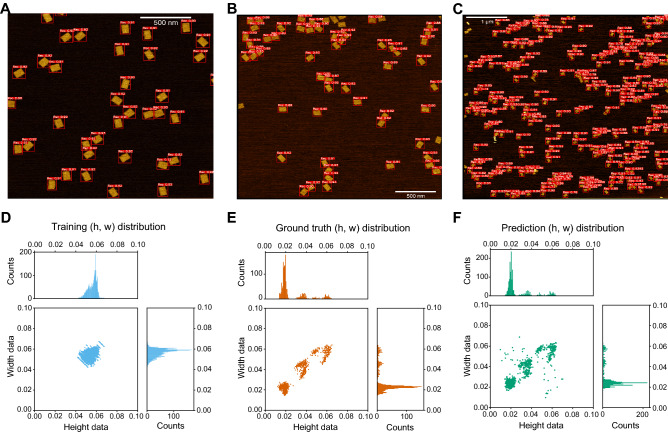
Model activation in response to high, moderate and low magnification. Image detection using the breadboard classifier at (**A**) high, (**B**) moderate, and (**C**) low magnification levels (see Supplementary Table 2 for pixel scale resolution). Scale bar in panels (**A**,**B**) = 500 nm and panel (**C**) = 1 μm. (**D**) Normalized bounding box height and width distribution of training annotations. Source images for the training data taken at a single magnification result in only a single visible data cluster. (**E**) Normalized bounding box height and width distribution of test set ground truth annotations. The three visible clusters correpsonds to data of high, moderate and low magnifications. (**F**) Normalized bounding box height and width distribution of the YOLOv5s model predictions. Three data clusters similar to the ground truth data are visible. The scattered data points correspond to noise stemming from erroneous predictions by the breadboard model.

**Figure 5:**
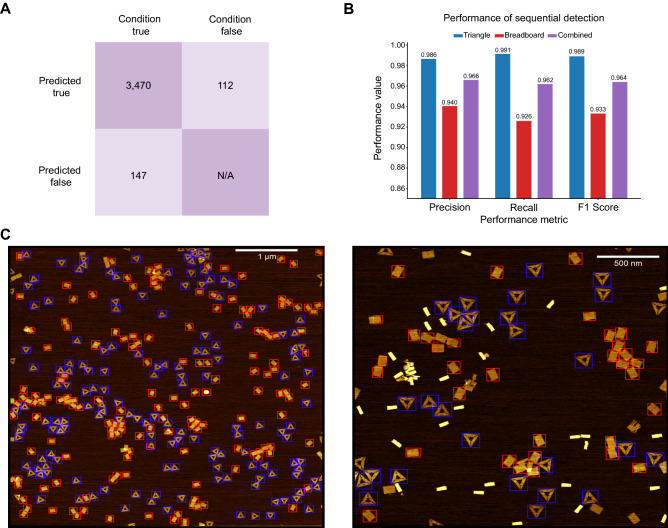
Combined performance with sequential detection. (**A**) Composite confusion matrix representing the combined performance of the triangle and breadboard models. (**B**) Precision, recall, and F1 scores corresponding to the triangle model, the breadboard model, and the combined performance. (**C**) Overlaid images post-detection using the triangle and breadboard classifiers. By running the classifiers sequentially, we can classify multiple geometries with high fidelity. Scale bar in panel (**C**) left = 1 μm and 500 nm for panel (**C**) right.

After obtaining the PyTorch weights files from the network training, the YOLOv5 detect function is utilized to make inferences on the test data (Fig. 2). The YOLOv5 detect function deploys the exported weights to predict the coordinates of potential DNA structure locations. The detect function outputs a copy of each input image with overlaid bounding box masks as well as a corresponding text file containing values for each predicted bounding box. Each box has five associated values: bounding box height, bounding box width, center x-coordinate, center y-coordinate, and confidence. The object bounding box refers to a rectangular box, which surrounds an object specifying its location and dimensions. By cross-referencing these bounding boxes with ground truth data, predictive performance is calculated. In practice, the YOLOv5 detect function performs inference across the entire test data set at once. Ergo, the performance of each test subset was calculated retrospectively. The inference performed by the triangle model took approximately 11.79 s while the breadboard took approximately 13.22 s. After performing inference, we used the ground truth data to analyze performance and curate the returned predictions (see Fig. 2). Upon analysis, the triangle model returned 1,996 predicted structures. Of these predictions, 1,969 were true positives (TPs), 27 were false positives (FPs), and 17 were false negatives (FNs) (Fig. 3E), see “Materials and methods”—Analysis subsection for definitions. These values correspond to a precision of 0.986, a recall of 0.991, and an F1 score of 0.989. Comparatively, the breadboard returned 1596 predictions, of which 1501 were TPs, 95 were FPs, and 120 were FNs (Fig. 3F), corresponding to a precision of 0.940, a recall of 0.926, and an F1 score of 0.933. By aggregating these results, the effective multiclass detection resulted in a total of 3592 predictions, of which 3470 were TPs, 122 were FPs, and 137 were FNs (Fig. 5A). This performance corresponds to a precision of 0.966 a recall of 0.962 and an F1 score of 0.964 (Fig. 5B). Once the overall performance was determined, the test subsets of interest were individually evaluated. We looked at the effect of including multiple structure geometries (Fig. 3), then the effect of varying degrees of magnification (Fig. 4), and lastly, we examined intra-image clustering (Supplementary Fig. 7). The predictive performance of each dataset is summarized in Table 2.

**Table 2:** Outline of the results of YOLOv5 object detection based on each test subset.

Test subset	YOLO model	TP	FP	FN	Precision	Recall	F1
Homogenous triangle	Triangle	1,338	19	17	0.986	0.987	0.987
	Breadboard	9	1	6	0.900	0.600	0.720
	Combined	1,347	20	23	0.985	0.983	0.984
Homogenous breadboard	Triangle	–	–	–	–	–	–
	Breadboard	789	58	40	0.932	0.952	0.942
	Combined	789	58	40	0.932	0.952	0.942
Heterogeneous	Triangle	631	8	0	0.987	1.000	0.994
	Breadboard	703	36	74	0.951	0.905	0.927
	Combined	1,334	44	74	0.968	0.947	0.958
Low Magnification	Triangle	979	16	6	0.984	0.994	0.989
	Breadboard	1,071	44	85	0.961	0.926	0.943
	Combined	2,050	60	91	0.972	0.957	0.964
Medium Magnification	Triangle	990	11	11	0.989	0.989	0.989
	Breadboard	218	4	35	0.982	0.862	0.918
	Combined	1,208	15	46	0.988	0.963	0.975
High Magnification	Triangle	–	–	–	–	–	–
	Breadboard	212	47	0	0.819	1.000	0.900
	Combined	212	47	0	0.819	1.000	0.900
Clustered Full	Triangle	646	8	10	0.988	0.985	0.986
	Breadboard	176	39	0	0.819	1.000	0.900
	Combined	822	47	10	0.946	0.988	0.966
Clustered 1 Quadrant	Triangle	93	1	1	0.989	0.989	0.989
	Breadboard	36	8	0	0.818	1.000	0.900
	Combined	129	9	1	0.935	0.992	0.963
Full Dataset	Triangle	1,969	27	17	0.986	0.991	0.989
	Breadboard	1,501	95	120	0.940	0.926	0.933
	Combined	3,470	122	137	0.966	0.962	0.964

*TP* True positives, *FP* False positives, *FN* False negatives,— Not available, see also “Materials and Methods—Analysis”.

Overall, the triangle model outperformed the breadboard model. In each case, the triangle model has an F1 score exceeding 0.98, where the breadboard tended to lag behind with lower 0.9 values. While the breadboard model displayed precision and recall values > 0.9 in most cases, the model displayed a marked decrease in precision for structures at high magnifications, this includes clustering images which were taken at a higher magnification.

## Discussion

Outside of model training, the potentially most time-consuming phase of this approach was the annotation of the initial training dataset. Assuming it takes, on average, about 10–15 s to manually annotate one structure, it would presumably take between 10 and 15 h to manually annotate our test dataset of 3592 data points. One advantage obtained by combining the use of labelImg and the YOLOv5 framework is that YOLOv5 is configured to output predictions formatted the same way as the training data annotations recognized by labelImg. Exploiting this fact allows the user to annotate a small fraction of their total dataset and quickly train a suboptimal YOLOv5 model. Then, this model can be used to rapidly annotate the rest of the dataset. An example protocol could involve annotating 200 structures, training a YOLOv5s model for about 1 h, then performing inference on the remainder of the unannotated dataset. Although this suboptimal network will be activated with a wide margin of error, a significant fraction of the predictions will be correct and data curation is much less time consuming than data annotation. We estimate these methods cut the upfront data annotation time at least in half. This technique also touches on the key point that the protocol is self-refining. Once a model is established, every instance a structure is identified, that prediction can be curated and then combined with the existing training set. Scheduled periodic retraining(s) on aggregate training sets could serve to boost performance.

Our first subset analysis focused on the issue of model activation in response to geometric specificity. We found for each model, activation was highly constrained to the specific target geometry. Each model was robust enough to suppress activation when presented with alternate geometries and partially formed structures. Here, test images were broken into three groups corresponding to a specific geometric distribution. The homogeneous triangle and homogenous breadboard groups consisted of a single geometry, while the heterogeneous group featured images of breadboard + triangle, and breadboard + triangle + nanotube (Fig. 3). In summary, both models show high F1 scores (> 0.90) when making predictions on both homogenous and heterogeneous data (Table 1). Interestingly, when utilizing our breadboard model to perform inference on the homogenous triangle data, we observed activation in two of the images. This was unexpected because, in theory, these are collective images of a homogeneous triangle sample and should result in no predictions returned by the breadboard model. However, upon further inspection, we discovered 15 contaminating breadboards, 9 of which were positively identified by the model. We speculate the source of this contamination arose during AFM sample preparation, specifically from the preparation of mica from block materials between samples; mica cleavage is imperfect and occasionally result in fissures that might allow samples to contaminate unexposed layers. Though unintentional, this behavior exemplifies a key advantage of automated structure detection. Minor instances of contamination can be identified in situations that manual analysis might have missed. An interesting observation is the consistently higher performance of the triangle model (Fig. 3E) compared to the breadboard (Fig. 3F). When it comes to the detection of a specific structure geometry, we observe a 5.5-fold decrease in FPs and a 4.4-fold decrease in FNs from the triangle model when compared to the breadboard. This could potentially be attributed to the inherent geometric differences, *i.e.,* the triangle due to its concatenated nature has six determinate angles as compared to four for the breadboard, or due to the increased amount of training data used to fit the triangle model. Alternatively, the physical properties of each structure may lead to deformations hindering efficient identification. For example, flexibility in the breadboard may lead to distortions reflected in changing edge length proportions. While this subtle change may be relatively imperceptible to a human, these slight changes may impact prediction confidence dropping it below the acceptable threshold. Furthermore, the monovalent salt content of the imaging buffer can cause shrinking, swelling or degradation depending on the concentration. However, these are speculations and would require further in-depth analyses. In any case, this variability emphasizes the importance of robust data augmentation strategies.

In addition to geometric sensitivity, we also address the issue of model activation in response to images with varied magnification. For the models, robust structure generalization to different size perspectives is an essential feature, as AFM experiments often include imaging with varying degrees of magnification and/or resolution. These findings indicate that each model fit, using data of a single magnification, can effectively generalize new perspectives and make accurate predictions at each magnification evaluated. To demonstrate this, test set images were included with varied magnifications. Upon plotting annotation bounding box heights and widths for the source dataset, a single cluster for both the breadboard and triangle training sets was observed (Fig. 4D & Supplementary Fig. 1). Comparatively, when plotting ground truth bounding box heights and widths, two distinct clusters for the triangle (Supplementary Fig. 3) and three clusters for the breadboard were seen (Fig. 4E). These clusters represent DNA structures in low, medium, or high magnification images (Fig. 4A–C). After performing inference, we clearly observe these same clusters reflected in the predicted bounding boxes for both triangle (Supplementary Fig. 5) and breadboard models (Fig. 4E). Upon quantifying the performance (Table 1) we see that in each instance both models have relatively high predictive performance (F1 ≥ 0.90). This result indicates the YOLO models can make accurate detection on different levels of magnification. Once more it was observed that the triangle model outperformed the breadboard. Another interesting result is the marked decrease in precision of the breadboard at the high magnifications. To boost inference performance for the breadboard detection, the image input size was increased to 1600 square pixels. Increasing the input image size and in turn resolution during inference incurs less computational cost compared to during training. This suggests image input size modulation for detection as a simple method of optimization.

Finally, we explored the effect of intra-image data clustering on model activation. Under certain conditions, DNA origami can have a tendency to aggregate on AFM image substrates^50^. In these instances, it would be critical to ensure both high density regions as well as intra-image spatial distribution of data does not adversely impact model predictions. For example, in a model fit with data uniformly distributed throughout the images, would a test set with data points constrained to a single image quadrant result in more false positive predictions compared to a four-quadrant distribution? In order to assay each model’s sensitivity to this data clustering, a subset of 10 test images was used for each geometry. Half of these 10 images are full in that data points appear in all four quadrants of the image (Supplementary Fig. 7). While the other 5 images are copies with data retained only in the lower right-hand quadrant and the rest of the pixels set to black (Supplementary Fig. 7). As seen in Table 1, the triangle model once again significantly outperforms the breadboard model. One interesting aspect of this data set is the significant decrease in precision of the breadboard model. The fact that the precision drops in both the full and 1 quadrant images indicates that the decreased performance is not a result of the data clustering. One theory as to the source of this decline is that the full images and thus their 1 quadrant counterparts are part of the highest magnification set. The breadboard seems to perform poorly when confronted with the higher magnification data and this could be a confounding variable. One speculation as to why this might be the case could stem from the augmentation strategies we employ, specifically the rotational augmentations. When we rotate the pixel data of the image, the image bounding box is sometimes expanded in order to prevent data point loss (see Supplemental Discussion). However, ignoring potential rotational effects, each model’s values of quality metrics are consistent between the full and 1 quadrant images, supporting our hypothesis that spatial clustering will not have a significant erroneous effect on our YOLOv5 model activation. In addition to single quadrant images there are also regions of high-density structure deposition through the test set. In these areas the spaces between the structures area are minimized and in some cases two structures may be touching. This sort of scenario does not seem to negatively affect the performance of the detector. For example, in the instances where two triangles are concatenated along one side, the model almost always identifies two triangles instead of a singular rhombus, another commonly used DNA architecture^55^.

Although each model is designed and trained only to detect a single architectural class, by automatically running the models sequentially, we can effectively attain multiclass architecture detection (Fig. 5C). For example, by combining the results of triangle and breadboard models, we can generate valuable statistics across an entire dataset rapidly. In total, 3,470 structures were correctly identified in a matter of 25.01 s, approximately 138 identifications per second (Fig. 5A). The combined performance of both classifiers can be quantified to a precision of 0.966, a recall of 0.962, and a F1 score of 0.964 (Fig. 5B). The true value of this approach may lie in its ability to be extrapolated to include an arbitrary number of classes for detection. Attempts at YOLO multiclass detection models to classify both triangle and breadboard were made. Difficulties, such as diametric opposition when optimizing the triangle vs breadboard (*i.e.,* when optimizing for one structure the performance of the other would suffer) were observed. We found the best strategy was simplifying to an additive single class object detection strategy. In theory, in adopting this strategy, a continuously expanding portfolio of single class detection models of common DNA origami architectures can be created. Once established, the field can begin to distribute models and standardize AFM data curation of DNA nanostructures. For example, it is currently difficult to give an unbiased interpretation of these models’ performance, as to our knowledge there are no DNA origami or AFM image benchmark datasets available publicly. This work may thus represent an initial step towards this goal.

The implementation of dCNNs for DNA nanostructure classification potentially offer improvements over manual classification in both speed and fidelity in certain instances. By this we mean to recognize that there are alternative methods of accelerating DNA nanostructure identification and sorting. For example, some methods use ML to enhance AFM operation and image acquisition process while using MATLAB^51,52^ and other scripting languages for structural identification. However, the current study distinguishes itself from other methods by eliminating reliance on certain criterion for consistent identification. For example, user-defined input variables such as pixel intensity threshold values, convolution kernel sizes, or pixel areas of a singular structural unit which may dramatically change in response to experimental conditions. Scripting requires these values be explicitly input where in neural networks optimal values are learned through training rather than defined. Additionally, the YOLO’s larger available input parameter space enables for higher probability of reliable detection across image sets captured with non-uniform experimental parameters (physical and computational) compared to what is possible with user defined inputs. In this sense the current study is not a technical innovation in ML nor a scientific innovation in DNA origami; rather it is a verified field test of an innovative solution to a common problem in the DNA origami field through the application of ML. The core goal of this study is not to present an AI platform to universally outperform all other DNA origami identification and sorting tools. But, instead to submit a new type of tool which may, in certain instances, be better suited to researchers’ needs while also being amenable to modification and appropriated as necessary. Furthermore, we simply wish to contribute to the ever-expanding toolbox of bio-AFM software, which is in need of direct community investment.

An interesting avenue of future study is using this system to examine dynamic structures, geometries which can have different projections, or architectures labeled with nanoparticles. It would be easy to incorporate these aspects into our current experimental framework. Consider for example a dynamic DNA origami which can be in either an “open” state or a “closed” state depending on reaction conditions. Depending on the goals of the operator, these two projections can be annotated in training as separate classes and then detected individually to determine reaction yield. Alternatively, the two different projections could be annotated as a single class in which case the model would be fit to correlate both projections to a single class and determine total DNA structures present. An alternative approach also being considered is to couple the YOLO output to a secondary CNN, which would analyze structures on a per unit basis. For example, using a DNA rectangle with four available nanoparticle binding sites, an image set can be captured, and the YOLO network can be utilized to identify the bounding box coordinates of each fully formed DNA rectangle. Then, using the bounding box coordinates, each DNA object can be programmatically clipped from the larger images. The clipped images can then be stacked and input into the secondary CNN which will analyze the structures on a per unit basis and evaluate which binding sites are occupied, what is the labeling efficiency, defect analysis, etc. For example the CNN utilized by Green et al.^53^ for defect analysis in DNA origami using DNA-PAINT would be an ideal candidate to use as a secondary CNN.

Using this approach, we were able to cut the time required for AFM image annotation from hours to less than 30 s. Furthermore, the proposed method is not subject to operator dependent variability and thus is of high repeatability. Additionally, the YOLO CNN method eliminates the need to recalibrate user defined variables, in response to varying experimental parameters. However, this does not detract from YOLO’s reproducibility as performing inference on a dataset with specific weights will always yield the same results unless the detection parameters are changed. Ultimately, this addresses a pertinent problem to DNA nanotech and research in general, *i.e.,* the problem of experimental replication. Many of the assembly and measurement protocols in the field of DNA nanotech can be variable. Furthermore, DNA origami can be composed of hundreds of individual strands, of which around 5% are often missing in “fully formed” structures; thus, it is difficult to define a specific threshold for classifying structures as fully formed vs partially formed. The misinterpretation of this data may result in over- and under-estimation of the fidelity of structural assembly in various studies. In these cases, well-trained CNN models with transferrable weights can be utilized to standardize AFM image analysis and thus eliminate the bias present in manual image analysis. It also does not require much computational cost as training times of 2–3 h are modest for the performance achieved here. This framework, and the YOLOv5 model in general, is highly scalable to large datasets; this is demonstrated by the relatively low training times required for our dataset. Thus, we propose this approach as a viable one for future development of automated systems for DNA origami analysis using AFM and perhaps even TEM images.

## Materials and methods

### DNA structures

The designs and staple strand sequences used in this work that determine the assembly of DNA origami sharp triangle^15^, rectangular breadboard^54^, and 30-helix nanotube^48^, as shown in Fig. 1, were reported previously. All single-stranded (ss) DNA oligonucleotides (staple strands) were purchased from Integrated DNA Technologies (IDT, Coralville, IA), and the scaffold strand (M13mp18 circular ssDNA, 7249 nucleotides (nt)) was purchased from Bayou Biolabs (Harahan, LA). For assembly, all DNA origami mixtures contained 20 nM of the scaffold strand and 100 nM of each staple strand in 0.5 × Tris buffer (TBE, 44.5 mM Tris, 44.5 mM boric acid, 1 mM EDTA, pH 8.3) with 12.5 mM MgCl_2_. The thermal annealing protocols used to guide self-assembly varied slightly for each design. For assembly, the sharp triangle mixture was heated to 85 °C and cooled to 4 °C at a rate of − 0.5 °C/min; the breadboard mixture was heated to 70 °C for 5 min, then cooled to 10 °C at a rate of − 0.1 °C/min; the nanotube mixture was heated to 85 °C for 5 min, cooled from 85 to 60 °C at − 0.2 °C/min, from 60 to 25 °C at − 0.067 °C/min, and from 25 to 4 °C at − 0.2 °C/min. After annealing, each structure was purified from excess staple strands by poly(ethylene glycol), PEG, precipitation as described^54,55^. Utilizing UV–Vis absorbance spectroscopy, specifically the DNA absorbance at 260 nm, each structure was rehydrated to 10 nM origami in 0.5 × TBE with 12.5 mM MgCl_2_ or 50 mM HEPES (N-(2-hydroxyethyl)piperazine-N′-(2-ethanesulfonic acid), pH 7.5) with 9 mM MgCl_2_. It should be noted that several AFM images of the DNA origami sharp triangle are of samples that were pre-filtered and imaged using protocols reported in^15^.

### AFM imaging

All images used were taken from an in-house archive of experimental results. Therefore, exact imaging protocol may differ slightly. Below we outline our general imaging protocol. Before imaging, DNA origami were diluted to 1 nM in 0.5 × TBE with 8 mM MgCl_2_. AFM imaging was performed on a fast-scan AFM by JPK Instruments (Germany) under AC fast imaging mode (liquid) with USC-F0.3-k0.3 AFM tips from NanoWorld (Neuchâtel, Switzerland). On a segment of freshly cleaved mica (0.9 cm diameter; Ted Pella Inc.) mounted to a magnetic puck, 15 μL of DNA origami solution was deposited and allowed to adsorb for 5 min. The surface was then washed twice with 100 μL of 0.5 × TBE with 8 mM MgCl_2_, filtered with a 0.2 μm syringe filter, deposited onto the mica and wicked off with the corner of a folded lint-free lab wipe. The sample was then transferred to the AFM stage and 100 μL of imaging buffer was deposited on the mica. For samples of the sharp triangle and breadboard, the imaging buffer was identical to the rinsing buffer. For any samples containing the nanotube, the imaging buffer included 15 mM MgCl_2_ to improve the stability of nanotubes on the mica. A 25 μL droplet of imaging buffer was deposited on the AFM tip, then the AFM tip mount was lowered into the sample buffer to create a liquid "chamber" for imaging. AFM topography images of between 1 × 1 μm^2^ and 5 × 5 μm^2^ were acquired with 1000–2000 pts/line and lines/scan at a scan rate of 3–6 Hz. Images were leveled by mean plane subtraction and flattened with row-wise alignment using Gwyddion^56^, a free SPM data analysis software, or JPK Data Processing software.

### Data preparation and processing

See Fig. 2 for a schematic overview of the data preparation, annotation, augmentation, training, inference, analysis, and curation. Raw AFM images were annotated using the open-source data annotation graphical user interface (GUI) tool “labelImg” (https://github.com/tzutalin/labelImg.git). LabelImg was installed from the python package index (PyPI) using pip. Using the labelImg GUI, bounding boxes were drawn around all fully formed DNA structures. Each bounding box encompassed the entire structure. Structures that were at any point intersecting with the image border were excluded. Each structure annotation was automatically saved in YOLO format to a corresponding text file.

Data augmentation tools (Supplementary Table 1) were implemented in Python (v3.7 and higher) using the OpenCV (v4.5.1) library. Data augmentation is a commonly used technique in deep learning-based object detection pipelines to generate more regularized training data. Access to large datasets is usually required to train a CNN model, which is not always feasible or practical to produce data sets of this magnitude experimentally. Thus, data augmentation, a process that takes currently available training datasets and generates slightly modified copies, represents an alternative method of achieving this goal. Each source image underwent four different augmentation transformations to create a final training set. The first three augmentation functions maintained the image's orientation and only modified pixel intensities. To accomplish this, the source images were input to a function that generated a copy of the original image with the color channels converted to grayscale. Next, the source images were input into a function that generated two copies where one had image contrast scaled up by a factor of 3 and the other had image contrast scaled down by a factor of 0.75. Here, a total of 3 new transformed images had been generated for each source image, thus the total image dataset would be 4n where n is the number of source images. This data set, of size 4n, was used as the validation set. To further augment the training images, each image from this 4n image set was input to a transformation function which generated a copy of the image rotated by a random angle θ, such that 1° < θ < 360°. This process was repeated 5 times, each with a different randomly selected θ. In total, the resulting dataset consisted of 20n unique images which were used as the training set. No processing or transformations of any kind were applied to the test set, which only contained raw AFM images exclusive from those in the training set. See Supplementary Table 1 for a link to download the full test set and source images. This data augmentation protocol was used for both the triangle and breadboard datasets.

### YOLOv5s training and prediction

The YOLOv5 object detection codebase was cloned from the master branch of the Ultralytics YOLOv5 repository^57^. For both the triangle and breadboard, a YOLOv5s model was trained using the train.py function for 1,000 epochs with an image size of 1280 and batch size 10. Only the default training hyper-parameters were used. The hyper-parameters, as well as the resulting trained weights, are available for viewing and download (see Supplementary Table 1). These weights were then used to perform inference on the test dataset which had been excluded from model training. Inference was performed using the detect.py function with an image size of 1280 for the triangle and 1600 for the breadboard. For the triangle, the confidence threshold and intersection over union (IoU) threshold were set to 0.80 and 0.50, respectively. For the breadboard, the confidence and IoU were 0.55 and 0.3, respectively. For reference, the IoU is a measure of the overlap between bounding boxes. The greater the overlap, the greater the IoU value, where perfectly overlapping boxes would have an IoU of 1. Setting an IoU threshold helps to eliminate multiple bounding boxes around the same object. The confidence score on the other hand measures the probability a given bounding box contains and object of interest. The threshold is set in order to constrain the model to only output predictions with a high probability of representing an object. A Jupyter notebook interactive walkthrough of training and inference is available for download as well (see Supplementary Table 1). All training and inference were performed using hardware accelerators rented from the Google Colaboratory cloud computing service.

### Analysis

The resulting inference images and predictions were analyzed using a combination of MATLAB and Python. The MATLAB code returned a confusion matrix indicating the number of true positives (TP), false positives (FP), and false negative (FN) predictions using manually curated annotations as ground truth data. The Precision, Recall, and F1 scores reported for model inference were calculated from TP, FP, and FN according to the following equations:

1 $$Precision = TP/(TP+FP)$$

2 $$Recall=TP/(TP+FN)$$

3 $$F1 = 2{*}\left(\frac{Precision*Recall}{Precision+Recall}\right)$$

Precision is often referred to as the positive predictive value and quantifies the proportion of true positive predictions (TP) from all condition predicted positive (TP + FP). Recall, or sensitivity, quantifies the proportion of true positive predictions (TP) from all condition positives (TP + FN). For a single measurement of model performance, the F1 score, the harmonic mean of the precision and recall, was also reported. All code is freely available for viewing and download; a link is provided in Supplementary Table 1.

## Supplementary Information

Supplementary Information.

## Data Availability

All data augmentation tools, written in Python, are freely available under the MIT license (https://github.com/mchirib1/Origami_Structure_Detection). The “You Only Look Once” version 5 (YOLOv5) object detection framework, developed by Ultralytics and written in Python, is also freely available under a GPL-3.0 license (https://github.com/ultralytics/yolov5). Accompanied with this publication are two Jupyter notebooks which are interactive implementations of both the augmentation tools and YOLOv5 training and detection. Also accompanied with this publication is a spreadsheet map to the URL location of important project components (source data, trained weights, model structures, etc.), excluding the fully augmented training and validation sets. All raw and processed data can be made available upon reasonable request. The total project electronic dataset equals approximately 2.2 GB total.
