# Supplementary material for: Rapid DNA origami nanostructure detection and classification using the YOLOv5 deep convolutional neural network
Source: Sci Rep. 2022 Mar 9;12:3871. doi: 10.1038/s41598-022-07759-3 (PMC8907326; doi:10.1038/s41598-022-07759-3)
Supplement: Supplementary file 1 — Supplementary Information. [file 41598_2022_7759_MOESM1_ESM.docx]

**Supporting Information**

**Rapid DNA Origami Nanostructure Detection and Classification Using the YOLOv5 Deep Convolutional Neural Network**

Matthew Chiriboga^1,2^, Christopher M. Green^1^, David A. Hastman^1, 3^, Divita Mathur^1,4^, Qi Wei^2^, Sebastían A. Díaz^1^, Igor L. Medintz^1*^, Remi Veneziano^2*^

1 – Center for Bio/molecular Science and Engineering Code 6900, U.S. Naval Research Laboratory, Washington, DC 20375, USA

2 –Department of Bioengineering, Volgenau School of Engineering, George Mason University, Fairfax, VA 22030, USA

3 – Fischell Department of Bioengineering, A. James Clark School of Engineering, University of Maryland, College Park, MD 20742, USA

4 – College of Science, George Mason University, Fairfax, VA 22030, USA

*Corresponding Authors

Email: Igor.Medintz@nrl.navy.mil, rvenezia@gmu.edu

**Contents:**

**Supporting Methods and Discussion Page**

**Rotational Augmentation Discussion S2-3**

**Table S1. Project Component Directory S3**

**Figure S1. Triangle Training Data Distributions S4**

**Figure S2. Breadboard Training Data Distributions S5**

**Figure S3. Triangle Ground Truth Data Distributions S6**

**Figure S4. Breadboard Ground Truth Data Distributions S7**

**Figure S5. Triangle Prediction Data Distributions S8**

**Figure S6. Breadboard Prediction Data Distributions S9**

**Figure S7. Clustered Image examples S10**

**Figure S8. Loss Convergence Plots S11**

**Table S2. Test Image Sizes/Resolutions S12-13**

**Figure S9. Rotated Expanded Bounding Box S14 Figure S10. Rotated Cropped Bounding Box S15 Figure S11. YOLO Image Resizing S16**

**Supplemental Discussion**

**Rotational Augmentation Discussion**

While analyzing specific subset populations of the test data, we observed high values of quality metrics detecting objects at low magnifications. Additionally, we observed the lowest performance stemming from the high magnification data. This indicates to us the model has generalized better to identify smaller rather than larger DNA structures. We speculate phenomenon could stem from the utilized methods of augmentation. Specifically, we think the rotational augmentation function which manipulates the image size introduces some scaling augmentation when loading the data into the YOLO training. This could potentially be contributing to the high-performance metrics for the low magnification images in comparison to the high magnification images.

When we rotate the pixel data of the image, the image bounding box is sometimes expanded **(Figure S9)** in order to prevent cropping out training data points **(Figure S10)**. For example, when the data is rotated by 45° **(Figure S9B)** the bounding box is expanded encapsulate the entire rotated image. Conversely, when the image data is rotated 90° or near 90° (**Figure S9A)** there is little, or no expansion required because an orthogonal rotation still fits ideally into the original square bounding box. These represent expansion boundary conditions, where rotations by non-orthogonal multiples of 45° (*i.e*., 45°, 135°, 225°, 315°) require the most expansion and orthogonal rotations (*i.e.,* 90°, 180°, 270°) require no expansion. Throughout the data augmentation process the pixel resolution of each nanostructure data point remains constant and only the size of the image is modified. However, this effect becomes impactful when training the YOLO model. Specifically, because training is computationally expensive the input images are downsized. For example, in this study YOLO was set to train on 1,280 by 1,280 pixel images. This is resizing of the augmented training data is non-trivial because although each of the inner images in **Figure S9** are the same dimensions and resolution, but the overall bounding boxes are expanded differently dependent on the magnitude of rotation. Therefore, during training when the images are all scaled to the 1,280 square pixels, the inscribed image containing the training data points will not always be scaled down by a constant factor factor.

For an illustrative example, see **Figure S11**. Briefly, a single source image is rotated 45° and 360°. Since 360° is a full rotation, there is no change to the bounding box and it remains at 1,664 by 1,664 pixels. The 45° rotation requires the bounding box to be expanded to 2,352 by 2,352 pixels in order to encapsulate the entire inner image and prevent cropping as seen in **Figure S10**. The expanded bounding box (2,352 by 2,352 pixels) is larger than the unexpanded box (1,664 by 1,664 pixels) by a factor of 1.4. It should be noted here that the inner image of the rotation is still 1,664 by 1,664 pixels meaning the data points in the inscribed image and the 360° rotated image are of the same scale and resolution. Then during YOLO training each image is scaled down to 1,280 by 1,280 pixels. Since the rotated image needs to be scaled down more to reach the 1,280 by 1,280 limit, the 360° rotation and the inscribed rotated image will no longer be of the same scale. In fact, the inner image of the 45° rotation is now 906 by 906 pixels. Given the linear nature of the transformations this makes logical sense since both bounding boxes are now 1,280 by 1,280 pixels. The inscribed rotated image (906 by 906 pixels) is now smaller than the fully rotated image (1,280 by 1,280 pixels) by a factor of 1.4.

Put into context of our observed results, we interpret this as a slightly confounding variable. The rotations introduce slight downscale augmentation which may boost the low magnification performance giving it a comparative edge over the high magnification. This may be an important consideration to keep in mind when using this method. The results suggest that a best use practice would be to include training structures at the highest magnification planned on being used. This would prevent the network from necessarily having to rely solely on generalization to higher magnification data. Practically, this also makes sense as low magnification data would allow a larger structure count to be captured and analyzed for more robust statistical analysis.

| **Index** | **Name** | **Link** |
| --- | --- | --- |
| **YOLOv5** |  |  |
| 1 | YOLOv5 Repo | https://github.com/ultralytics/yolov5 |
| 2 | YOLOv5s Model Structure | https://github.com/ultralytics/yolov5/blob/master/models/yolov5s.yaml |
| 3 | Training Script | https://github.com/ultralytics/yolov5/blob/master/train.py |
| 4 | Detection Script | https://github.com/ultralytics/yolov5/blob/master/detect.py |
| **In House Code** |  |  |
| 1 | Augmentation Jupyter Notebook | https://github.com/mchirib1/Origami_Structure_Detection/tree/master/misc-analysis/jupyter_notebooks |
| 2 | Training and Detection Jupyter Notebook | https://github.com/mchirib1/Origami_Structure_Detection/tree/master/misc-analysis/jupyter_notebooks |
| 3 | MATLAB Analysis Script | https://github.com/mchirib1/Origami_Structure_Detection/tree/master/misc-analysis/matlab |
| 4 | Augmentation Utilities | https://github.com/mchirib1/Origami_Structure_Detection/tree/master/utils |
| **Data Sets** |  |  |
| 1 | Test | https://github.com/mchirib1/Origami_Structure_Detection/tree/master/datasets/test |
| 2 | Triangle Source | https://github.com/mchirib1/Origami_Structure_Detection/tree/master/datasets/triangle_train |
| 3 | Breadboard Source | https://github.com/mchirib1/Origami_Structure_Detection/tree/master/datasets/breadboard_train |
| 4 | Triangle Training | Available Upon Request |
| 5 | Triangle Validation | Available Upon Request |
| 6 | Breadboard Training | Available Upon Request |
| 7 | Breadboard Validation | Available Upon Request |
| **Misc.** |  |  |
| 1 | PyTorch Trained Weights - Triangle | https://github.com/mchirib1/Origami_Structure_Detection/tree/master/datasets/pretrained_weights |
| 2 | PyTorch Trained Weights - Rectangle | https://github.com/mchirib1/Origami_Structure_Detection/tree/master/datasets/pretrained_weights |

**Table S1:** Project component download locations.


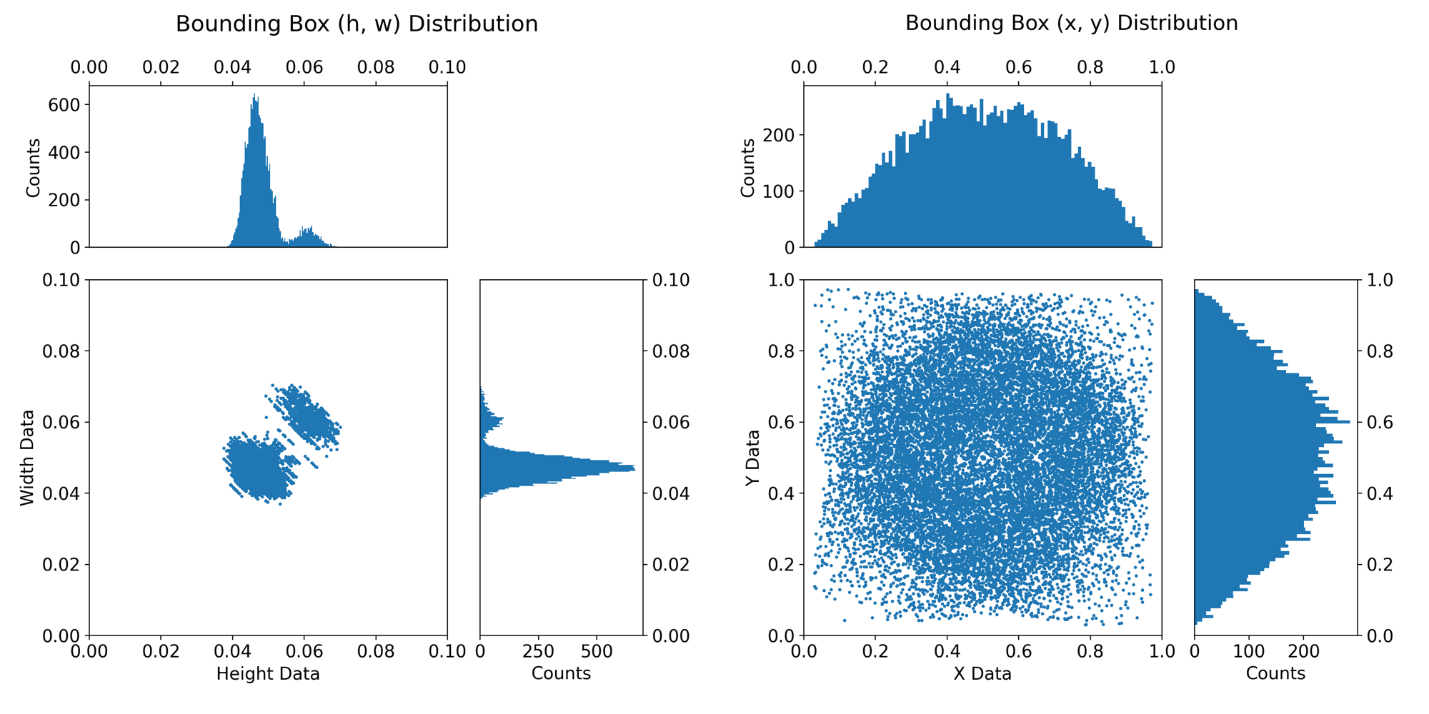


**Figure S1:** Triangle training annotation bounding box distributions. (Left) The plotted distribution of bounding box height and width data for the training annotations of the triangle structure. (Right) The plotted distribution of bounding box Cartesian coordinates based on the center x and center y data for the training annotations of the triangle structure. Data points here refer to the augmented training data.


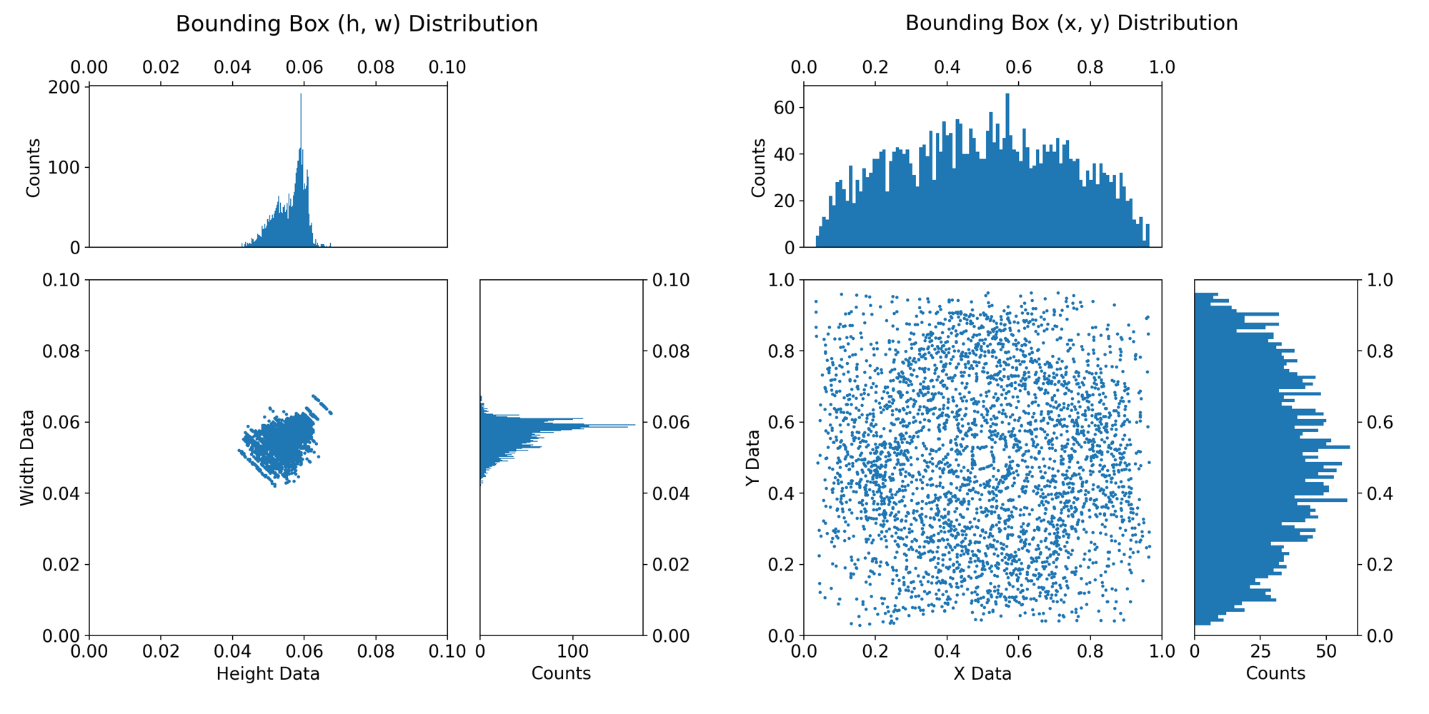


**Figure S2:** Breadboard training annotation bounding box distributions. (Left) The plotted distribution of bounding box height and width data for the training annotations of the breadboard structure. (Right) The plotted distribution of bounding box Cartesian coordinates based on the center x and center y data for the training annotations of the breadboard structure. Data points here refer to the augmented training data.


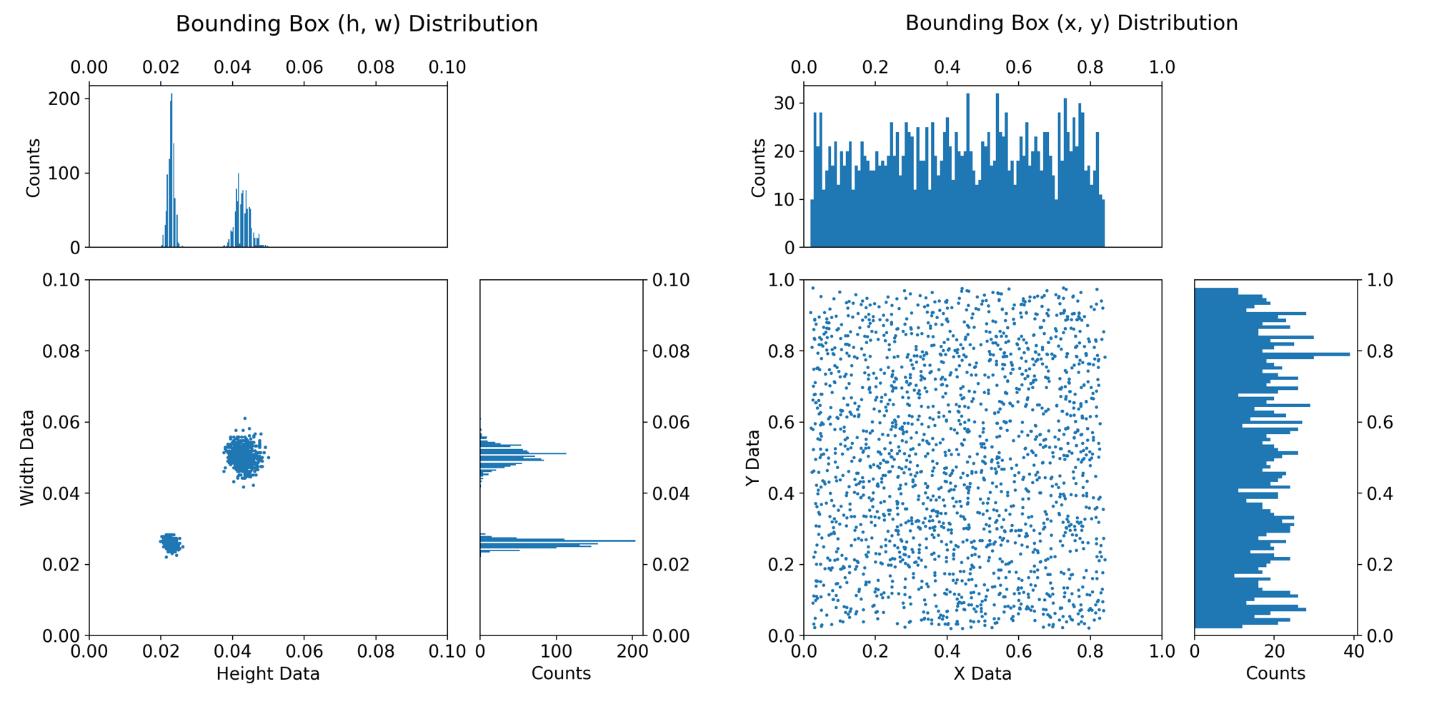


**Figure S3:** Triangle ground truth bounding box distributions. (Left) The plotted distribution of bounding box height and width data for ground truth annotations of the triangle structure. The red boxes correspond to data points of high and low magnifications. (Right) The plotted distribution of bounding box Cartesian coordinates based on the center x and center y data for ground truth annotations of the triangle architecture. The empty region indicated by the red arrow corresponds to the scale bar of the test AFM images. This scale bar is the reason no structures are present in this area.


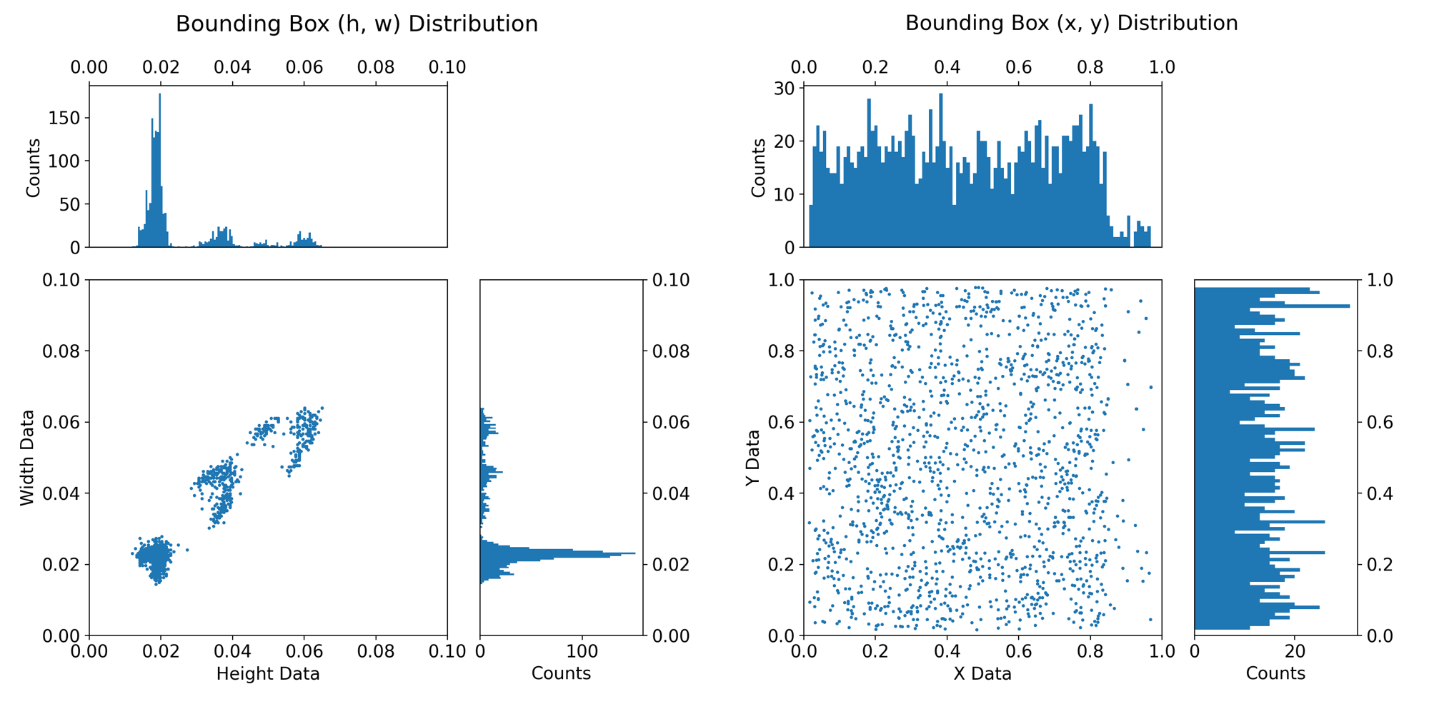


**Figure S4:** Breadboard ground truth bounding box distributions. (Left) The plotted distribution of bounding box height and width data for ground truth of the breadboard structure. The three red boxes correspond to data points of the three different magnifications. (Right) The plotted distribution of bounding box Cartesian coordinates based on the center x and center y data for ground truth of the breadboard structure. Some breadboard images lacked the reference scale bar which explains the amount of ground truth annotations in the region indicated by the red arrow.


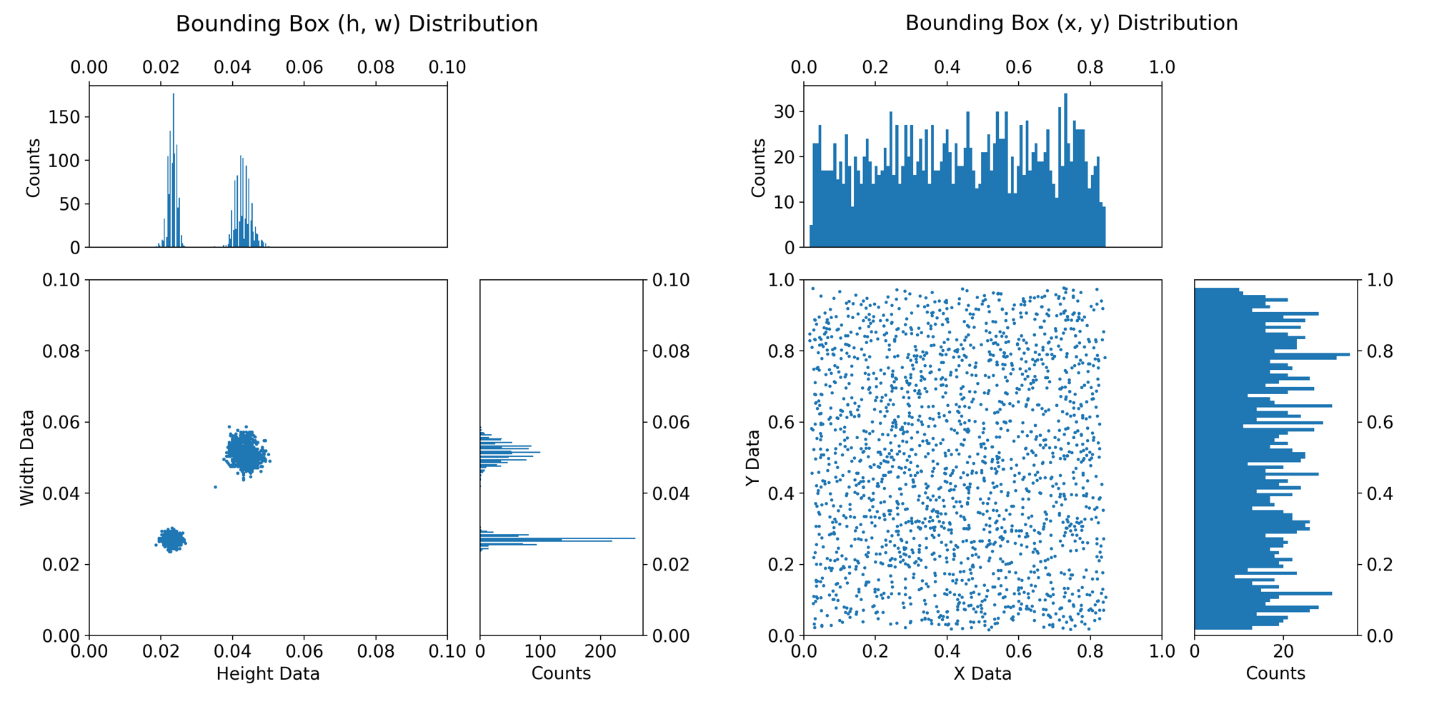


**Figure S5:** Triangle predicted bounding box distributions. (Left) The plotted distribution of bounding box height and width data for YOLOv5s predictions of the triangle structure. The red boxes indicate predictions corresponding to the two different magnifications. (Right) The plotted distribution of bounding box Cartesian coordinates based on the center x and center y data for predictions of the triangle structure.


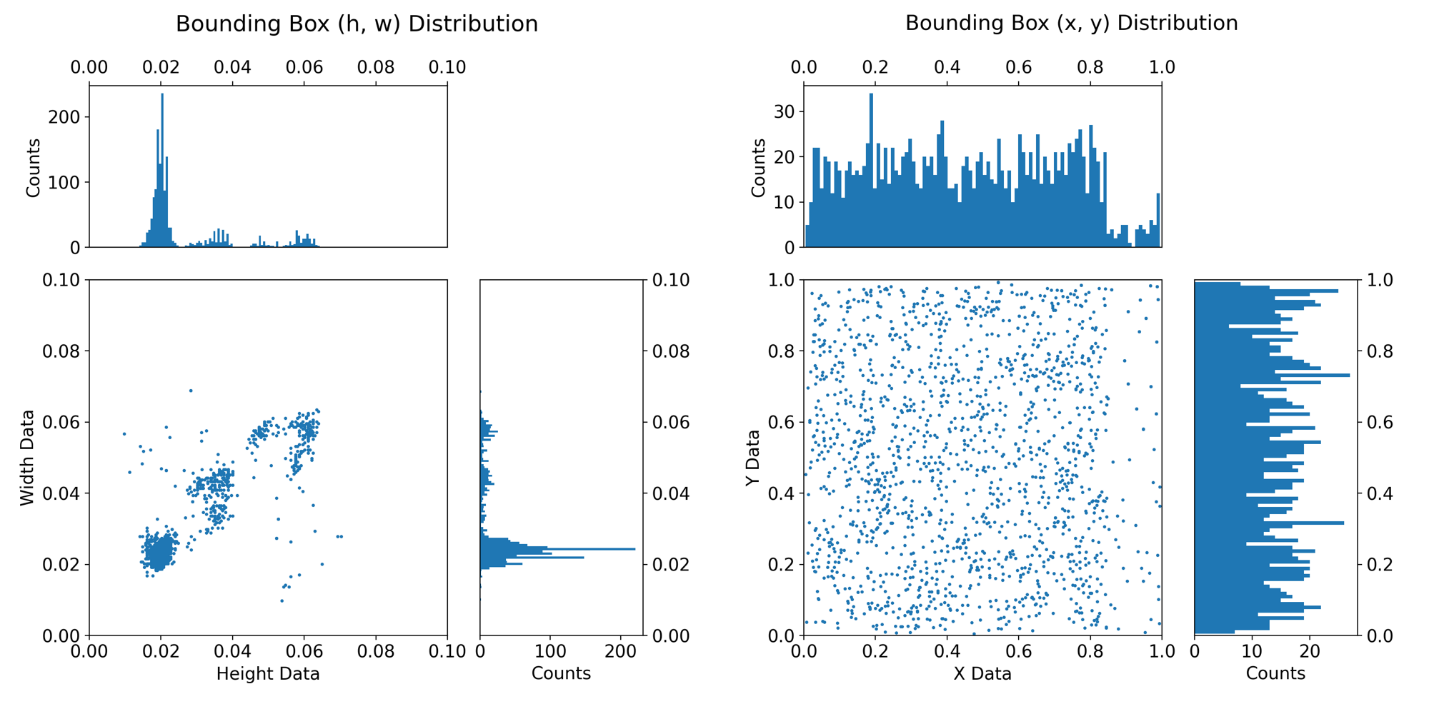


**Figure S6:** Breadboard predicted bounding box distributions. (Left) The plotted distribution of bounding box height and width data for YOLOv5s predictions of the breadboard structure. The red boxes indicate predictions corresponding to the three different magnifications. (Right) The plotted distribution of bounding box Cartesian coordinates based on the center x and center y data for predictions of the breadboard structure.


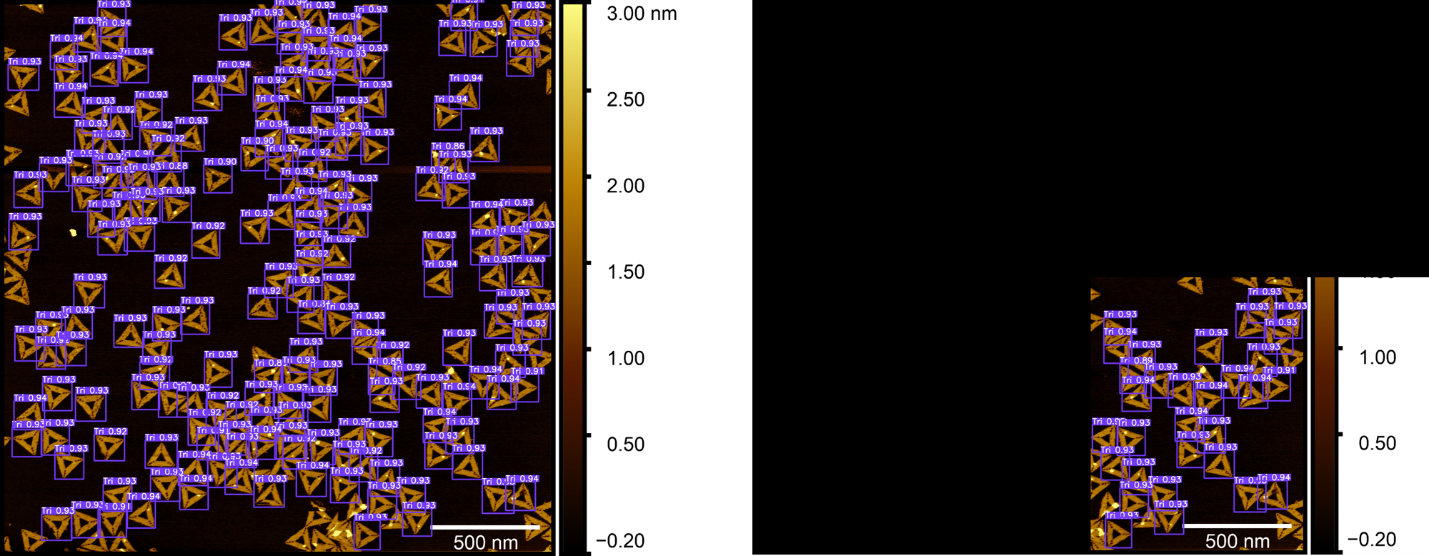


**Figure S7:** Example clustering images of DNA triangle. Example clustering subset image (left) and its corresponding 1 quadrant counterpart (right).

**
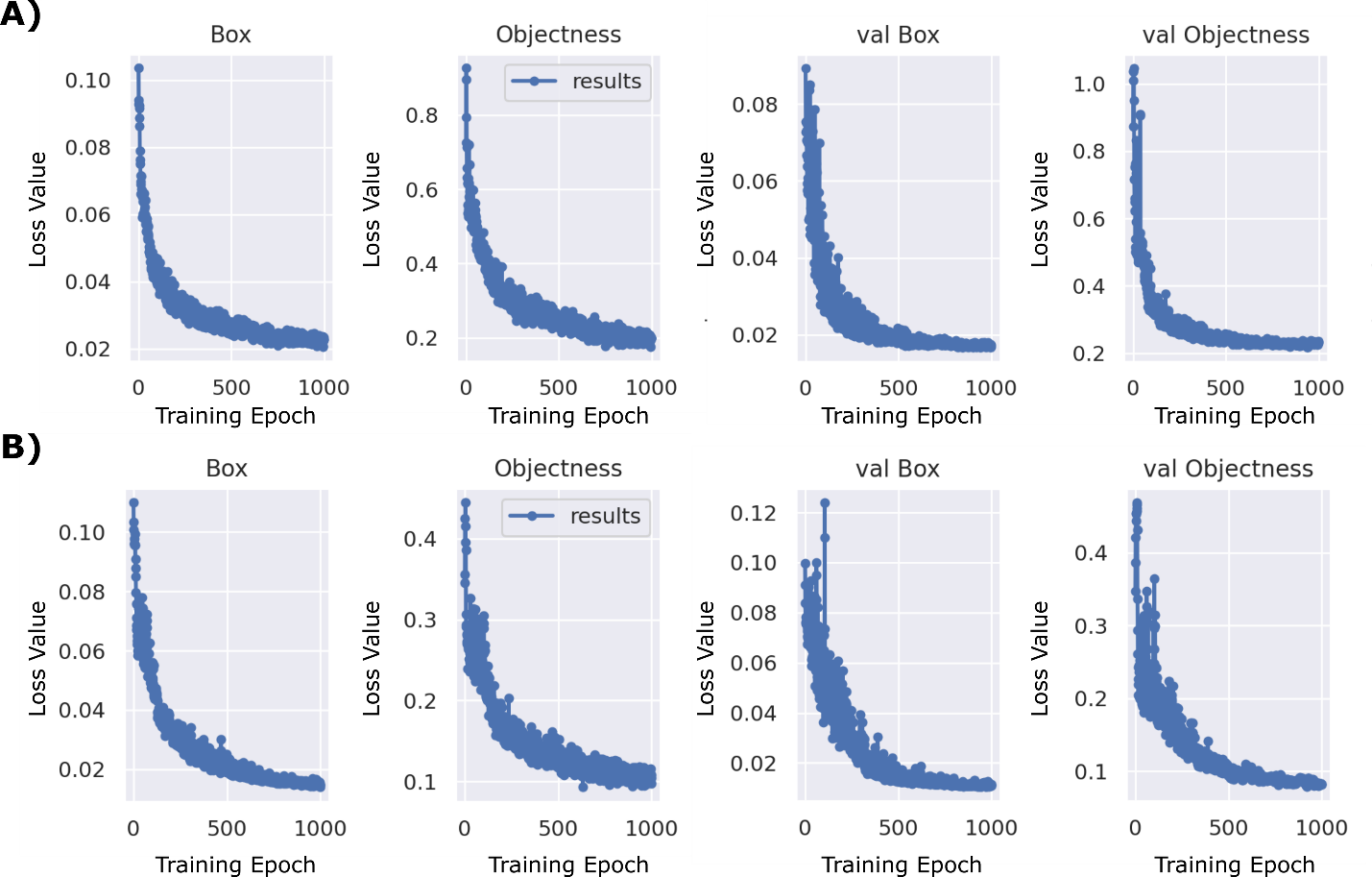
**

**Figure S8:** Loss convergence plots. Loss convergence of the **(A)** triangle and **(B)** breadboard model trainings. Box loss describes the model’s ability locate the center of an object and its ability to represent it with a predicted bounding box. Objectness refers to the model’s ability to produce bounding boxes in regions where an object exists with high probability.

| **Image** | **Resolution of 1600 px^2^ (px*nm^-1^)** | **Resolution of 1280 px^2^ (px*nm^-1^)** | **Magnification Group** |
| --- | --- | --- | --- |
| 2020-09-16_Mixed-sample-standard-0000.png | 0.5 | 0.4 | Medium |
| 2020-09-16_Mixed-sample-standard-0001.png | 0.5 | 0.4 | Medium |
| 2020-09-16_Mixed-sample-standard-0002.png | 0.3 | 0.2 | Low |
| 2020-09-16_Mixed-sample-standard-0003.png | 0.3 | 0.2 | Low |
| 2020-09-16_Mixed-sample-w-negative-standard-0000.png | 0.5 | 0.4 | Medium |
| 2020-09-16_Mixed-sample-w-negative-standard-0001.png | 0.5 | 0.4 | Medium |
| 2020-09-16_Mixed-sample-w-negative-standard-0002.png | 0.3 | 0.2 | Low |
| 2020-09-16_Mixed-sample-w-negative-standard-0003.png | 0.3 | 0.2 | Low |
| 2020-09-16_Rectangle-standard-0002.png | 0.5 | 0.4 | Medium |
| 2020-09-16_Rectangle-standard-0003.png | 0.5 | 0.4 | Medium |
| 2020-09-16_Rectangle-standard-0004.png | 0.3 | 0.2 | Low |
| 2020-09-16_Rectangle-standard-0005.png | 0.3 | 0.2 | Low |
| 2020-09-16_Triangle-standard-0000.png | 0.5 | 0.4 | Medium |
| 2020-09-16_Triangle-standard-0001.png | 0.5 | 0.4 | Medium |
| 2020-09-16_Triangle-standard-0002.png | 0.3 | 0.2 | Low |
| 2020-09-16_Triangle-standard-0003.png | 0.3 | 0.2 | Low |
| 3quad_test_rectangle_1.png | 0.8 | 0.6 | High |
| 3quad_test_rectangle_2.png | 0.8 | 0.6 | High |
| 3quad_test_rectangle_3.png | 0.8 | 0.6 | High |
| 3quad_test_rectangle_4.png | 0.8 | 0.6 | High |
| 3quad_test_rectangle_5.png | 0.8 | 0.6 | High |
| 3quad_test_triangle_1.png | 0.5 | 0.4 | Medium |
| 3quad_test_triangle_2.png | 0.5 | 0.4 | Medium |
| 3quad_test_triangle_3.png | 0.5 | 0.4 | Medium |
| 3quad_test_triangle_4.png | 0.5 | 0.4 | Medium |
| 3quad_test_triangle_5.png | 0.5 | 0.4 | Medium |
| test_rectangle_1.jpg | 0.8 | 0.6 | High |
| test_rectangle_2.jpg | 0.8 | 0.6 | High |
| test_rectangle_3.jpg | 0.8 | 0.6 | High |
| test_rectangle_4.jpg | 0.8 | 0.6 | High |
| test_rectangle_5.jpg | 0.8 | 0.6 | High |
| test_triangle_1.png | 0.5 | 0.4 | Medium |
| test_triangle_2.png | 0.5 | 0.4 | Medium |
| test_triangle_3.png | 0.5 | 0.4 | Medium |
| test_triangle_4.png | 0.5 | 0.4 | Medium |
| test_triangle_5.png | 0.5 | 0.4 | Medium |

**Table S2:** Test set image magnification size/resolution.


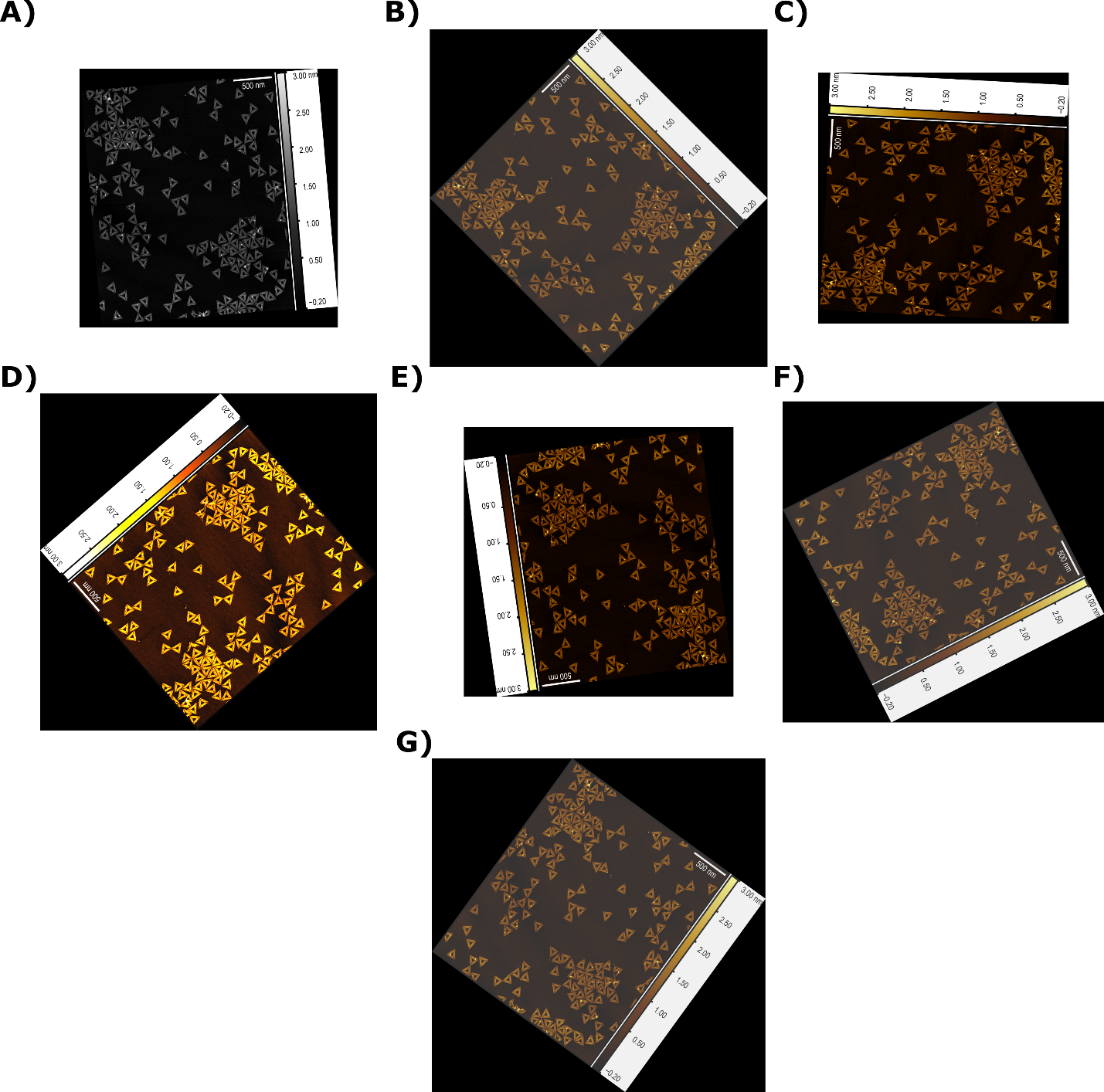


**Figure S9:** Rotational augmentation examples of a single source image with expanding bounding box. A single image augmented and then rotated counterclockwise **(A)** 5°, **(B)** 45°, **(C)** 87°, **(D)** 131°, **(E)** 188°, **(F)** 292°, and **(G)** 314°. In each rotation the bounding box is expanded with empty space to prevent data points from being cut off in as seen in **Figure S10**. The inner image of each rotation maintains constant pixel resolution and dimensions.


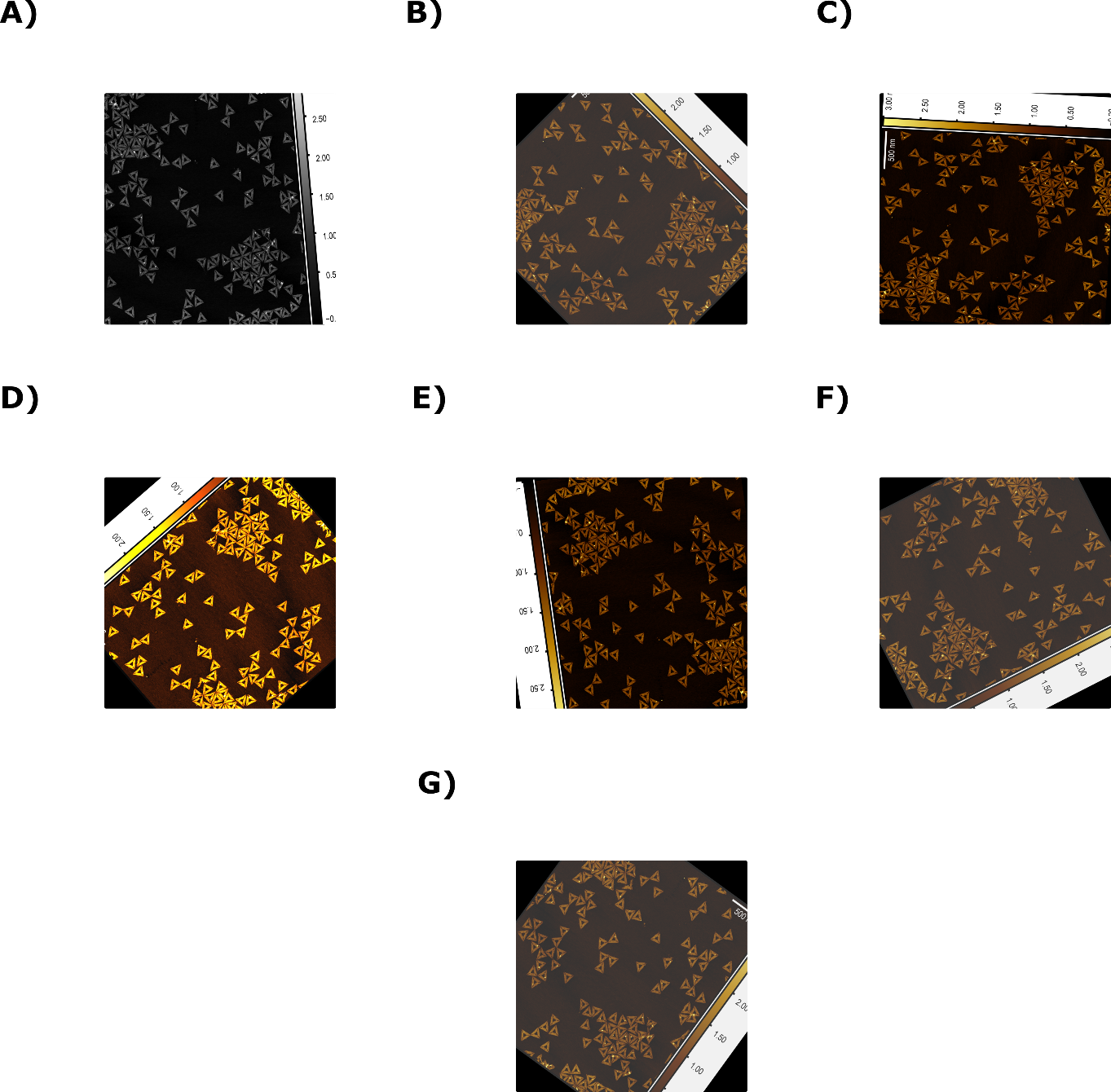


**Figure S10**: Rotational augmentation examples of a single source image with cropped bounding box. A single image augmented and then rotated counterclockwise **(A)** 5°, **(B)** 45°, **(C)** 87°, **(D)** 131°, **(E)** 188°, **(F)** 292°, and **(G)** 314°. In each rotation, everything outside of the original pixel of the original untransformed bounding box are kept which results in cropped corners and lost data points.


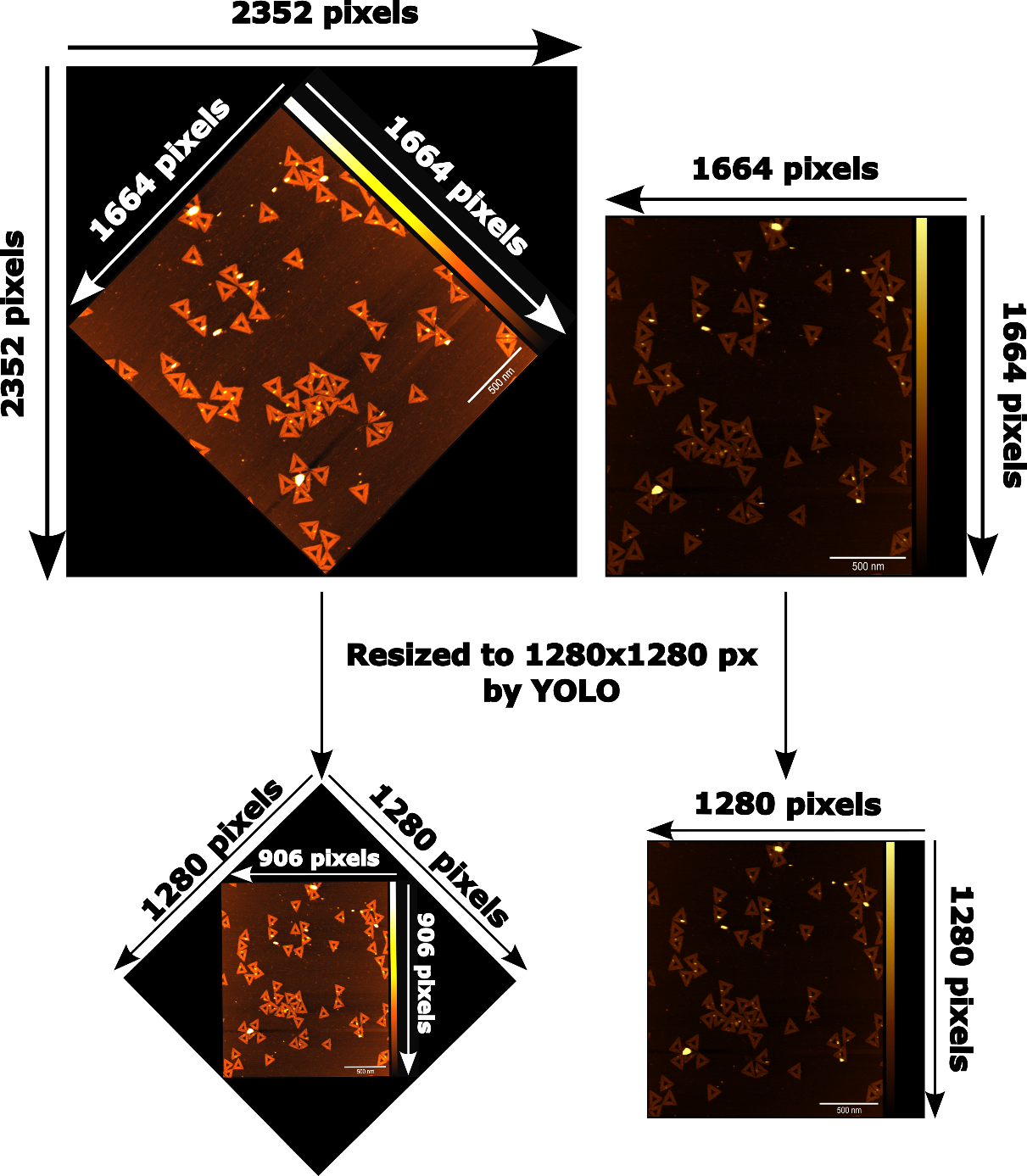


**Figure S11:** Rotated data input to YOLO and corresponding scaling. Examples of two different image rotations and the subsequent bounding box expansion which results in non-proportional scaling upon YOLO training. Top left and right show the pixel-by-pixel count of the full bounding boxes with the top left inner image being the same dimensions as the top right image. YOLO training results in rescaling the entire bounding box to the designated size and thus the bottom left inner image is scaled greater than the bottom right image.
